# In This Issue

**Published:** 2003

## ALCOHOLISM AND THE BRAIN: AN OVERVIEW

Alcoholism can affect a person’s brain and behavior in a variety of ways, ranging from simple “slips” in memory to permanent and debilitating conditions that result in a need for custodial care. The risk of alcohol-induced brain damage and related neurobehavioral deficits also varies from person to person and is influenced by factors such as age, gender, drinking history, and nutrition. In this overview, Drs. Marlene Oscar-Berman and Ksenija Marinkovic explain these risk variables and describe techniques that researchers use to study the effects of alcoholism on the brain and behavior. Neuroimaging techniques, for example, provide researchers with an inside look at the structure and activity of the living brain and enable them to more accurately assess alcohol’s effects. The authors explore the implications of this research for alcoholism treatment. They suggest that neuropsychological observations and structural and functional brain imaging results can be used to tailor treatment to individual patients and to more accurately monitor the course of treatment. (pp. 125–133)

## THE ROLE OF THIAMINE DEFICIENCY IN ALCOHOLIC BRAIN DISEASE

The vitamin thiamine plays a key role in the breakdown of carbohydrates in brain cells, as described by Drs. Peter R. Martin, Charles K. Singleton, and Susanne Hiller-Sturmhöfel. Without thiamine, cells cannot form vital brain chemicals such as neurotransmitters as well as other molecules that are essential for building proteins and DNA. The result may be the development of serious brain disorders, including Wernicke-Korsakoff syndrome. This condition, found primarily in alcoholics, occurs when a person fails to take in enough thiamine in his or her diet, when thiamine absorption from the gastrointestinal tract is compromised, or when thiamine is not used sufficiently by the cells. Not all alcoholics experiencing thiamine deficiency develop Wernicke-Korsakoff syndrome, which suggests that other influences (e.g., genetic factors) help to determine a person’s susceptibility to the effects of thiamine deficiency. In addition, some brain regions may be more sensitive to thiamine deficiency than others. (pp. 134–142)

## HEPATIC ENCEPHALOPATHY— A SERIOUS COMPLICATION OF ALCOHOLIC LIVER DISEASE

Brain damage in alcoholics can be a direct effect of alcohol’s action on the brain as well as a side effect of alcoholic liver disease. Dr. Roger F. Butterworth describes how persistent alcohol-related liver dysfunction leads to the accumulation of toxic ammonia and manganese in the blood and brain, leading to hepatic encephalopathy—a serious and potentially fatal brain disorder characterized by severe cognitive effects, psychiatric conditions, and motor disturbances. To prevent or ameliorate the consequences of hepatic encephalopathy, researchers and clinicians are exploring a variety of approaches, ranging from nutritional supplements to lower ammonia levels in the blood to liver transplantation. (pp. 143–145)

## USING MAGNETIC RESONANCE IMAGING AND DIFFUSION TENSOR IMAGING TO ASSESS BRAIN DAMAGE IN ALCOHOLICS

Research using brain imaging techniques has revealed that certain brain areas are often smaller in volume in people with a history of chronic alcohol dependence than they are in non-alcoholic subjects. This brain shrinkage as well as the disruption of fibers that carry information between brain cells (i.e., white matter) and impairment of associated cognitive and motor functions are examples of the brain damage that may occur as a result of excessive alcohol consumption. Drs. Margaret Rosenbloom, Edith V. Sullivan, and Adolf Pfefferbaum describe how imaging techniques can be used in patients to detect and quantify these abnormalities on both macrostructural and microstructural levels. Conventional structural magnetic resonance imaging (MRI) reveals the size, shape, and tissue composition of the brain and its constituent parts. Diffusion tensor imaging (DTI) reveals the integrity of white-matter tracts that link regions of the brain to one another. The authors point out that researchers using DTI to study alcoholics have been able to detect abnormalities in white matter not visible with conventional MRI. They suggest that DTI may be especially useful in elucidating the mechanisms that underlie the changes in the brain that occur with abstinence and relapse. (pp. 146–152)

## ALCOHOLISM AND HUMAN ELECTROPHYSIOLOGY

Researchers use electroencephalography (EEG), a technique for recording electrical signals from the brain, to evaluate brain function as it is happening. Studies using EEG, as well as event-related potentials (ERPs) and event-related oscillations (EROs), which measure brain activity in response to a specific stimulus, have shown that the brain activity of alcoholics and nonalcoholics differs in some characteristic ways. As Drs. Bernice Porjesz and Henri Begleiter explain, these differences are consistent with an imbalance in the brain’s state of being “excited” and “inhibited.” This imbalance in excitation–inhibition may place some people at greater risk of developing problems with alcohol. For example, long-term studies of childhood and adolescent precursors of adult alcohol abuse consistently identify a cluster of behavioral traits described as disinhibited, undercontrolled, impulsive, or aggressive, which significantly predict high levels of alcohol consumption or abuse. (pp. 153–160)

## POSITRON EMISSION TOMOGRAPHY—A TOOL FOR IDENTIFYING THE EFFECTS OF ALCOHOL DEPENDENCE ON THE BRAIN

One important approach to identifying alcohol’s effects on the brain, particularly after long-term consumption leading to alcohol dependence, is to look at various brain structures and their activities in living patients. Drs. Dean F. Wong, Atul Maini, Olivier G. Rousset, and James Robert Brašić describe positron emission tomography (PET) technology, which allows investigators to visualize specific brain structures and their metabolic activities. With this approach, researchers have been able to identify areas where shrinkage of brain tissues occurs as well as specific brain chemical (i.e., neurotransmitter) systems whose functions are altered by chronic exposure to alcohol. In addition to providing a better understanding of alcohol’s effects on the brain, PET technology offers a means of developing and assessing new medications to correct alcohol-induced deficits in certain brain chemicals. PET also makes it possible to identify patients who are most likely to benefit from specific interventions. (pp. 161–173)

## ALCOHOL AND THE DEVELOPING BRAIN: NEUROANATOMICAL STUDIES

Prenatal exposure to alcohol may lead to changes in brain anatomy (i.e., neuroanatomy) as well as to impaired cognitive and behavioral function. Drs. Wei-Jung A. Chen, Susan E. Maier, and James R. West, and Mr. Scott E. Parnell review findings from human and animal studies outlining the neuroanatomical changes associated with prenatal alcohol exposure. These changes include significantly smaller brain sizes as well as alterations in cellular activity. The authors explain how current neuroimaging technology provides powerful tools for assessing the damaging effects of fetal alcohol exposure on the brain and for exploring the relationship between behavioral dysfunction and brain damage. The authors also describe promising techniques to prevent or reverse alcohol-induced neuroanatomical changes, including pharmacological treatments and complex motor training. (pp. 174–180)

## MALE AND FEMALE SENSITIVITY TO ALCOHOL-INDUCED BRAIN DAMAGE

Are women more vulnerable than men to alcohol-induced brain damage? Although research shows that women may be at higher risk than men of developing many of the medical consequences of alcohol use, such as alcohol-induced liver disease, the findings on gender and alcohol-induced brain damage are inconclusive. Dr. Daniel W. Hommer reviews the current research, which shows that male alcoholics generally have smaller brain volumes than nonalcoholic males. Only a few studies have compared brain structure in alcoholic men and women, and those have had mixed results. The author notes that other factors, such as age, also affect gender differences in brain structure, and those factors must be considered when comparing brain damage between alcoholic men and women. (pp. 181–185)

## WHAT HAPPENED? ALCOHOL, MEMORY BLACKOUTS, AND THE BRAIN

Alcohol interferes with people’s ability to form new long-term memories, and the magnitude of memory impairment increases with increasing alcohol use. In this article, Dr. Aaron M. White summarizes research on alcohol-induced memory dysfunction, with particular focus on alcohol-induced blackouts—periods for which a person is unable to remember events that occurred while he or she was intoxicated. In addition to the overall amount of alcohol consumed, a rapid rise in blood alcohol level—which would occur, for example, when a person drinks on an empty stomach—make blackouts more likely. Other evidence indicates that some people may be more sensitive than others to the memory-impairing effects of alcohol. Finally, blackouts are more common among social drinkers, including college drinkers, than was previously assumed. This article examines brain mechanisms posited to underlie alcohol-induced memory impairment, including disruption of hippocampal and frontal lobe function. (pp. 186–196)

## ALCOHOL, NEURAL STEM CELLS, AND ADULT NEUROGENESIS

For years it was believed that each person was born with a finite number of brain cells, which could not be re-created. Recent research demonstrates, however, that stem cells in some brain regions are capable of giving rise to new cells (a process known as neurogenesis) throughout life. In this article, Drs. Fulton T. Crews and Kim Nixon examine two questions concerning alcohol and adult neurogenesis: To what extent are neurogenesis in the adult brain and the risk for alcoholism governed by similar factors? And how, if at all, do alcohol use and alcoholism affect adult neurogenesis? Genetic and environmental factors that regulate neurogenesis overlap significantly with those involved in the development of alcoholism, the authors contend. Further, research indicates that heavy alcohol use can disrupt neurogenesis in adults, leading to long-term effects on brain structure and function. More moderate but chronic drinking appears to affect neurogenesis, possibly through a process similar to the neurodegeneration associated with chronic alcoholism. Several mechanisms may play a role in alcohol-induced disruptions in neurogenesis, including increases in stress hormone levels, disruption of cell-to-cell communication, and inhibition of cell growth. (pp. 197–204)

